# Lignans and Their Derivatives from Plants as Antivirals

**DOI:** 10.3390/molecules25010183

**Published:** 2020-01-01

**Authors:** Qinghua Cui, Ruikun Du, Miaomiao Liu, Lijun Rong

**Affiliations:** 1 College of Pharmacy, Shandong University of Traditional Chinese Medicine, Jinan 250355, China; duzi857@163.com (R.D.); 17862972322@163.com (M.L.); 2 Qingdao Academy of Chinese Medicinal Sciences, Shandong University of Traditional Chinese Medicine, Qingdao 266122, China; 3 Research Center, Shandong University of Traditional Chinese Medicine, Jinan 250355, China; 4 Department of Microbiology and Immunology, College of Medicine, University of Illinois at Chicago, Chicago, IL 60612, USA

**Keywords:** lignans, antivirals, mechanism, drug development

## Abstract

Lignans are widely produced by various plant species; they are a class of natural products that share structural similarity. They usually contain a core scaffold that is formed by two or more phenylpropanoid units. Lignans possess diverse pharmacological properties, including their antiviral activities that have been reported in recent years. This review discusses the distribution of lignans in nature according to their structural classification, and it provides a comprehensive summary of their antiviral activities. Among them, two types of antiviral lignans—podophyllotoxin and bicyclol, which are used to treat venereal warts and chronic hepatitis B (CHB) in clinical, serve as examples of using lignans for antivirals—are discussed in some detail. Prospects of lignans in antiviral drug discovery are also discussed.

## 1. Introduction

**Figure 1:**
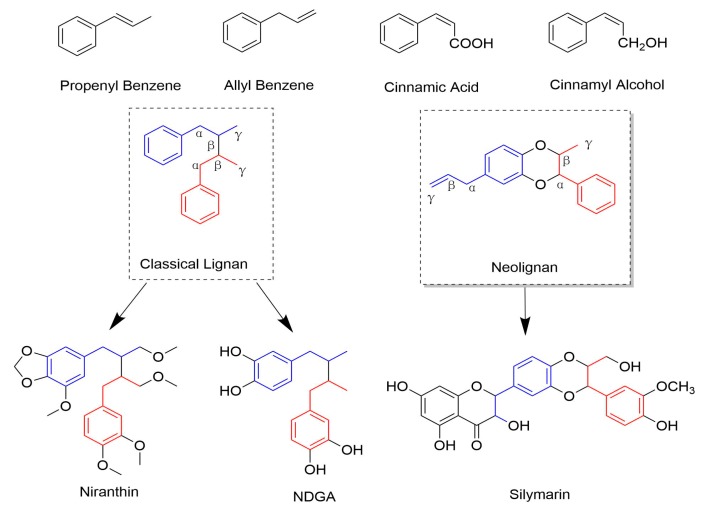
The monomers and this classification of lignans.

Lignans are a large group of naturally occurring compounds that are derived from the shikimic acid biosynthetic pathway [1]. Structurally, Lignans contain a basic scaffold of two or more phenylpropanoid units [2], and the monomers forming lignans are cinnamic acid, cinnamyl alcohol, propenyl benzene, and allyl benzene. When the molecular linkage of monomers occurs between positions β-β′ (also referred to as an 8-8′), these compounds are designated as “classical lignans”. In contrast, the compounds are grouped into “neolignans” if the main structural units are coupled in any other way (non β-β′ linkage). Figure 1 shows the monomers and the classification. Neolignans have more varied structures than classical lignans.

Lignans are widely distributed in the plant kingdom, and they exist in plant roots, rhizomes, stems, leaves, flowers, fruits, seeds, xylem, and resins. Plants, such as the *Lauraceae* family, especially the genera of *Machilus*, *Ocotea*, and *Nectandra* are rich sources of lignans. Additionally, *Annonaceae*, *Orchidaceae*, *Berberidaceae*, and *Schisandraceae* family contain a large number of constituents of lignans and neolignans [3,4,5]. Up to date, lignans are found in over 70 families in plant kingdom, and more than 200 classical lignans and 100 neolignans have been characterized [6]. They are usually present as dimers, but some of them are trimers or tetramers. Most of the lignans in plants are in a free state, while some of them can combine with glycon and form glycosides and other derivatives.

With such structural diversity of lignans being discovered, it is not surprising that many attractive pharmacological activities of the lignan family, such as antitumor [7], antioxidant [5], antibacterial [8], immunosuppressive [9], and antiasthmatic properties [10] were reported. Pertinent to this review, many lignans have been identified with antiviral activities [11]. Tubulin binding, reverse transcriptase inhibition, integrase inhibition, and topoisomerase inhibition are included as the reported mechanisms of antiviral activities [12]. Here, we will highlight the antiviral activities and mechanisms of action (MOA) of different lignans and their derivatives.

## 2. Antiviral Effect and MOA

Lignans display a vast structural diversity due to the numerous potential coupling modes of the phenoxy radicals [13]. As mentioned above, they can be grouped into two subclasses: classical lignans and neolignans. Next, we will discuss the antiviral lignans and possible MOA, according to different subclasses, and then summarize them in Table 1 at the end of this section.

### 2.1. Classical Lignans

**Figure 2:**
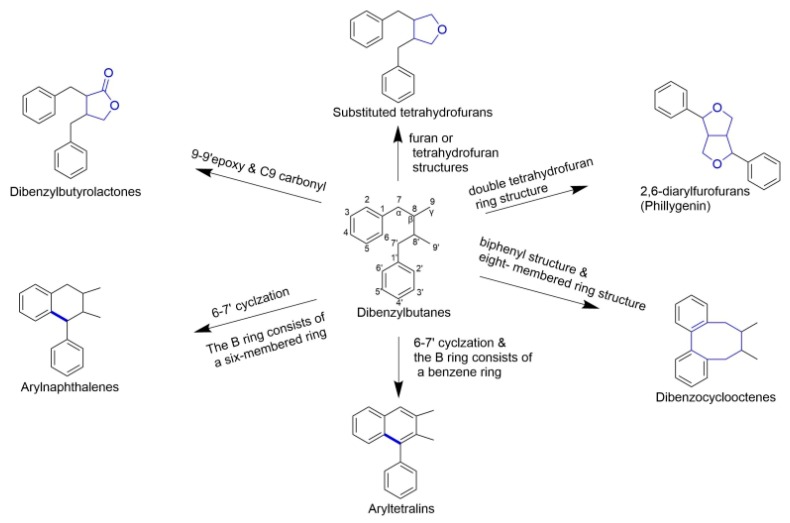
Relationships between different classical lignans. It depicts the basic mother nucleus structure of different subtypes of classical lignans, the main structural feature of this subclass is the β-β′ linkage. Dibenzylbutane (central position) is the basic structure of classical lignan, other subtypes of lignans derive from this structure with different chemical reactions.

The classical lignans contain dimeric structures that are formed by a β-β′-linkage between two phenyl propane units, some of them with a different degree of oxidation in the side-chain and a different substitution pattern in the aromatic moieties. They can be classified into six major subtypes—dibenzylbutanes, dibenzylbutyrolactones, arylnaphthalenes/aryltetralins, substituted tetrahydro-furans, 2,6-diarylfurofurans, and dibenzocyclooctadienes [6,14]. Figure 2 illustrates the structures and relationships among them.

#### 2.1.1. Dibenzylbutanes

**Figure 3:**
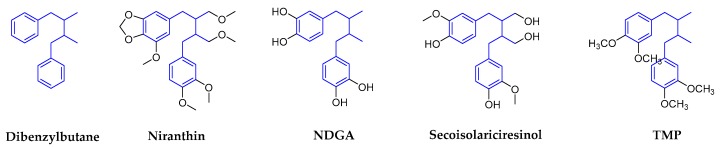
Structures of dibenzylbutanes and corresponding compounds.

Dibenzylbutanes, which are also known as simple lignans, are the simplest classical lignans, which are the only lignan subtype without being cyclized. They are phenylpropane dimers that have a β-β′ linkage. Dibenzylbutane lignans also show an increased diversity due to multiple possible oxidation states along the butane chain [15]. Niranthin, nordihydroguaiaretic acid (NDGA), and terameprocol (TMP), are the representative compounds with antiviral activity in this subclass, and Figure 3 shows their structures.

Niranthin. This compound was first isolated from *Phyllanthus niruri Linn*. (family Euphorbiaceae) [16], which has long been used in folk medicine for liver protection and anti-hepatitis B virus (HBV) in many Asian countries. Ray-L screened 25 compounds from *Phyllanthus* Species in vitro and niranthin showed the best anti-HBsAg activity among them [12]. When evaluated for the anti-HBV activity in vitro, niranthin was found to significantly decrease the secretion of HBsAg and HBeAg with IC_50_ values of 15.6 and 25.1 µM in the human HBV-transfected cell line HepG2.2.15, respectively. In vivo, niranthin treatment of the DHBV-infected ducklings significantly reduced the serum DHBV DNA, HBsAg, HBeAg, ALT, and AST. Mechanistic studies showed that niranthin inhibited not only DHBV DNA replication, but also HBV antigen expression, which suggests that niranthin acts as an anti-HBV agent through at least two or more targets [16].

NDGA was isolated from the leaves of *Larrea tridentata* (Zygophyllaceae); the plant was known as creosote bush, which has been traditionally used in folk medicine across different countries and regions for more than 50 different diseases [17]. It was reported that NDGA exerts beneficial effects on diverse diseases, like cancer, renal damage, Alzheimer’s disease, and other neurodegenerative pathologies [18,19,20]. At the molecular level, NDGA is a potent scavenger of reactive oxygen species [21]. NDGA has been identified to inhibit the replication of the related dengue virus (DENV); MOA showed that it inhibits DENV infection by targeting genome replication and viral assembly [22]. Moreover, NDGA showed the effect against hepatitis C virus (HCV), West Nile Virus (WNV), and Zika Virus (ZIKV) in vitro [23,24]. For influenza A viruses (IAV), NDGA can suppress the replication of IAV and the induction of cytokines, trypsin, and MMP-9, with improved animal survival [25]. See Table 1 for more details.

TMP is the shorter title of tetra-*O*-methyl nordihydroguaiaretic acid. It is a methylated derivative of NDGA and was also initially founded in the resin of the creosote bush [26]. As the derivative of NDGA, it was tested the antiviral effects against WNV and ZIKV simultaneously with NDGA; the results showed both compounds inhibited the infection of WNV and ZIKA, with good and similar IC_50_ values, and MOA was likely by impairing viral replication [24]. Meanwhile, Pollara showed that TMP inhibits poxvirus growth in vitro by preventing the efficient spread of virus particles from cell to cell [27]. Additionally, there were some reports regarding the antiviral activity of TMP against herpes simplex virus (HSV) and human immunodeficiency virus (HIV) [28,29]. Moreover, it was made into vaginal ointment for women with HPV-linked cervical intraepithelial neoplasia and it showed an excellent safety profile in Phase I/II trials [30].

Besides, Xu isolated four new lignans from the aerial parts of *Justicia procumbens* (Acanthaceae) and tested their activity against HIV-1. One of the new secoisolariciresinol dimethylether acetate exhibited anti-HIV-1 activity with an IC_50_ of 5.27 µM in vitro [31].

#### 2.1.2. Dibenzylbutyrolactones

**Figure 4:**
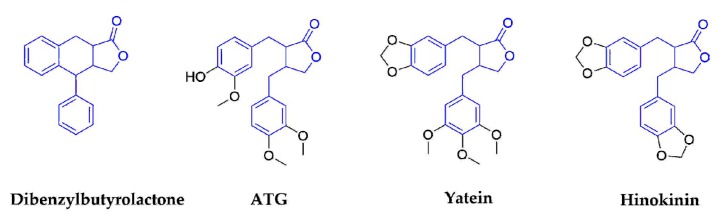
Structures of dibenzylbutyrolactone and corresponding compounds.

Dibenzylbutyrolactones, which are also known as lignans-β-β′-lactones(lignanolides), are based on dibenzylbutanes, with 9-9′epoxy and C9 carbonyl. Lignanolides are often found in the same plants as theirmonode- hydrogenated or didehydrogenated compounds and corresponding derivatives. The representative compounds with antiviral activities in this subclass are arctigenin (ATG), yatein, and hinokinin (Figure 4).

ATG was initially isolated from *Arctium lappa* L. (Compositae). So far, the research of antiviral activity has been mainly focused on IAV and HIV. The erythrocyte agglutination test showed that ATG can inhibit the replication of the IAV in vitro, and the inhibition was shown to be 100% with a concentration of 26.8 mM based on hemagglutination titer [32]. In vivo, ATG can reduce lung index, increase the survival rate of the infected mice, and induce the interferon levels of normal mice, which suggested that mechanistically ATG can induce the production of interferon [33]. ATG and its glycoside arctiin were also shown to be orally effective, but less than oseltamivir, the results suggested that it is a good choice of the combined arctiin with oseltamivir for IAV in immunocompromised mice that were infected with IAV [34]. Additionally, ATG strongly inhibited the expression of protein P17 and P24 of the HIV-1 in vitro. MOA showed that it targets reverse transcription [35]. Studies on the structure-activity relationship (SAR) showed that: (1) the structure of lactones is necessary; and, (2) the number and arrangement of phenolic hydroxyl groups are very important for the activity of lignanolides [36].

Yatein was isolated from *Chamaecyparis obtuse* (Cupressaceae). It could significantly suppress HSV-1 replication in HeLa cells without apparent cytotoxicity [37]. MOA showed that yatein can inhibit HSV-1 alpha gene expression, including the expression of the *ICP0* and *ICP4* genes, by arresting HSV-1 DNA synthesis and structural protein expression in HeLa cells [38].

Hinokinin was first isolated from the ether extract of *Chamecyparis obtusa* in 1933, and it was also found in different species of *Phyllanthus* (Euphobiaceae) [39], *Aristolochia* (Aristolochiaceae) [40], *Piper* (Piperaceae) [41], *Virola* (Myristicaceae) [42], *Linum* (Linaceae) [43], and so on. The anti-inflammatory, antimicrobial activities, and cytotoxicity of this compound have been extensively studied [44]. Meanwhile, it showed good antiviral activities against human HBV [12], HIV [45], SARS-virus (SARS-CoV) [46], and human cytomegalovirus (HCMV) [47]. The defects are all data from in vitro and no in-depth research on MOA.

#### 2.1.3. Arylnaphthalenes/Aryltetralins

**Figure 5:**
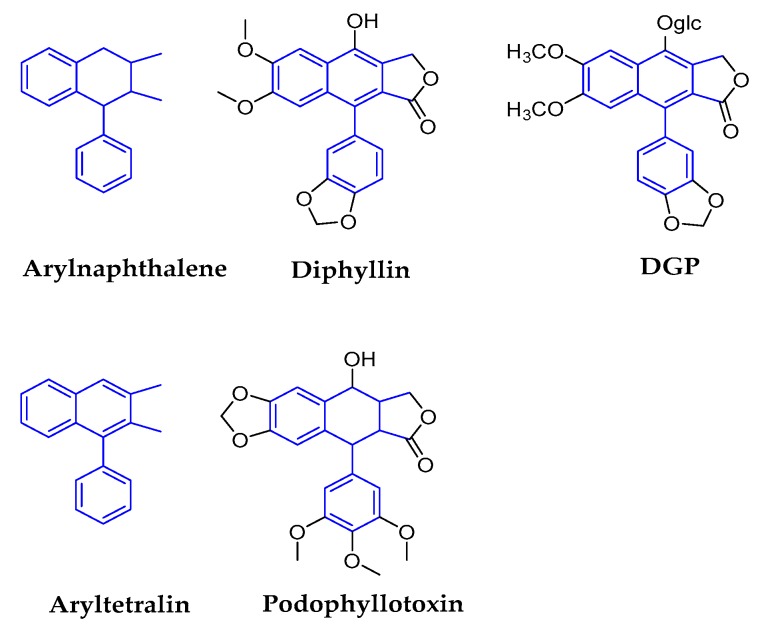
Structures of arylnaphthalene/aryltetralin and corresponding compounds.

The relationship between the arylnaphthalene and aryltetralin subclasses is of interest due to their deceptive structural similarities. Both of these compounds are based on dibenzylbutanes and formed by cyclization of six sites in one C6-C3 unit and seven sites in another C6-C3 unit. Their subtle structural difference lies in whether the B ring consists of a benzene ring or a six-membered ring. Arylnaphthalenes are also named benzene tetrahydronaphthalene, which means that the B-ring structure consists of six-membered rings. Aryltetralins are named benzene naphthalene, because the B-ring structure consists of benzene. The representative compounds of arylnaphthalene are diphyllin and 6-deoxyglucose-diphyllin (DGP), and podophyllotoxin represents aryltetralin (Figure 5).

Diphyllin is a natural component of plants with a naphthalene and one hydroxyl lignans [48]. It exists in *Haplophyllum alberti-regelii*, *H. bucharicum*, and *H. perforatum* (Rutaceae) [49]. It showed broad-spectrum antiviral activity as a potent vacuolar ATPase (V-ATPase) inhibitor [50]. For example, it blocked ZIKV infection in HT1080 cells with an IC_50_ of ~0.06 μM [51]; it also altered the cellular susceptibility to IAV through the inhibition of endosomal acidification, thus interfering with downstream virus replication [52]. There are more reports regarding the antiviral effect of glycosylated diphyllin.

DGP, which is also known as patentiflorin A, was first isolated from plant of *Justicia gendarussa* (Acanthaceae) [53]. As the glycosylated diphyllin, it exhibited anti-ZIKV activity both in vitro and *in vivo*, and it displayed broad-spectrum antiviral activity against other flaviviruses. MOA showed that DGP inhibits ZIKV fusion with cellular membranes and infection by preventing the acidification of endosomal/lysosomal compartments in the target cells [51]. Besides, it also displays potent activity against a broad spectrum of HIV strains with IC_50_ values in the range of 15–21 nM [54]; MOA showed that it acts as a potential inhibitor of HIV-1 reverse transcription [55].

Podophyllotoxin is one of the best-characterized lignans which is a type of aryletralin lignan lactone, and it was initially found in *Dysosmae Verspiellis Rhixoma* Et Radix or American mandrake or mayapple (all belong to family Berberidaceae) [3]. One of the research interests of podophyllotoxin is focused on anti-cancer activities [56,57]. Furthermore, it was first cited in 1942 as a topical treatment for venereal warts (Condyloma acuminatum), which is an ailment that is caused by papillomavirus [11]. The clinical randomized controlled trial data with 45 cases showed that podophyllotoxin 0.5% solution has a beneficial effect on anoenital warts and it is effective and safe for untreated anogenital warts in immunocompetent individuals [58].

#### 2.1.4. Substituted Tetrahydrofurans

**Figure 6:**
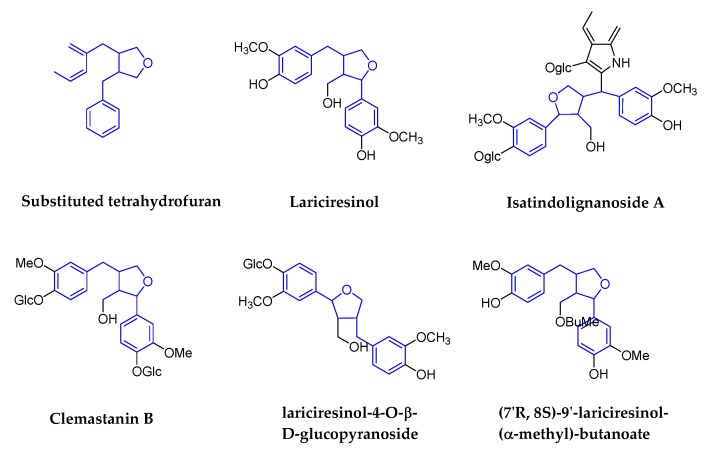
Structures of substituted tetrahydrofurans and corresponding compounds.

Substituted tetrahydrofurans are also designated as monoepoxylignans. It refers to the formation of furan or tetrahydrofuran structures that are based on dibenzylbutanes; the representative compounds are lariciresinol (LA) and the derivatives. Figure 6 shows the structures’ details. There are lots of traditional medicinal plants, such as *Patrinia scabra* Bunge (Caprifoliaceae) [59], *Stelleropsis tianschanica* (Rutaceae) [60], and *Rubia philippinensis* (Rubiaceae) [61] with ingredients of LA and the derivatives. Among them, the plant of *Isatis indigotica* Fort (Cruciferae) was the most studied because of the root.

The root of *Isatis indigotica* Fort is a very famous antiviral traditional medicine in China and is called *Radix Isatidis* (*Banlangen* in Chinese); during the prevalence of SARS in 2003, the traditional Chinese medicine products containing *Radix Isatidis* were once out of stock in China. So far, lots of derivatives with LA structure were isolated from *Radix Isatidis* and antiviral activities were demonstrated. For example, lariciresinol-4-*O*-β-d-glucopyranoside was shown to inhibit the IAV-induced pro-inflammatory response [62]; the underlying defense mechanism against IAV infection is from pharmacological actions on the immune system, signal transduction, cell cycle, and metabolism [63]; (*7′R*,*8S*)-9′-lariciresinol-(alpha-methyl)-butanoate showed a low amount of activity to anti-HIV-1 [64]; Isatindolignanoside A was shown to have antiviral activity against Coxsackievirus B3 (CVB3), with IC_50_ and SI values of 25.9 μM and >3.9, respectively [65]; Clemastanin B (*7S*,*8R*,*8′R*-(−)-lariciresinol-4,4′-bis-*O*-β-d-glucopyranoside), as the active ingredient of *Radix Isatidis*, it showed to inhibit different subtypes of human IAVs (H1N1, H3N2, and influenza B) [66].

#### 2.1.5. 2,6-Diarylfurofurans

**Figure 7:**
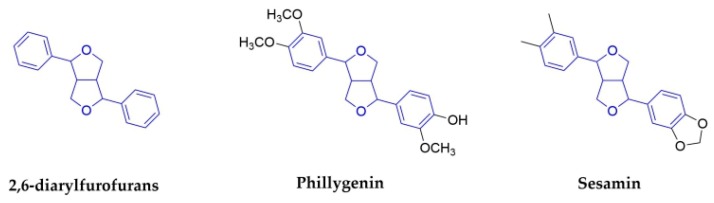
Structures of 2,6-diarylfurofurans and corresponding compounds.

2,6-diarylfurofuran, which is also known as bisepoxylignan, is a lignan with a double tetrahydrofuran ring structure, which is formed by two side chains of phenylpropanoid interlinked to form two epoxy structures. There are a few reports of these compounds on antiviral activities (Figure 7).

Phillygenin is the major active constituent of *Fructus Forsythiae* (Oleaceae). It can suppresses high glucose-induced lipid accumulation and it has antibacterial and antioxidant activities [56], and is could also be a potential therapeutic agent for alleviating inflammation [57]. Antiviral studies show that phillygenin has good protective effects against infections that are caused by IAV; it could reduce inflammation that is caused by IAV in vivo in the meanwhile [58].

Sesamin was isolated from the seeds of *Sesamum indicum* (Pedaliaceae). It has anti-inflammatory cytokines in human PBMCs that are induced by H1N1 [67]. However, there is no report demonstrating its direct anti-influenza activity.

#### 2.1.6. Dibenzocyclooctenes

**Figure 8:**
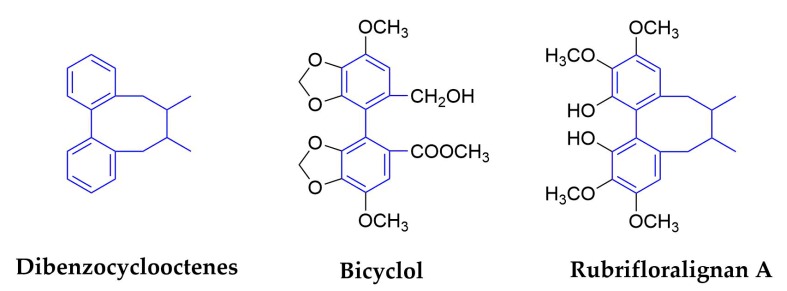
Structures of dibenzocyclooctene and corresponding compounds.

The structure of this subclass of lignans has not only biphenyl structure, but also an eight-membered ring structure synthesized by biphenyl and side chainring. Figure 8 shows the structures of dibenzocyclooctadiene and the corresponding compounds. So far, more than 150 lignans have been isolated and identified from more than 60 species of *Schisandraceae* family [68]. The reason of dibenzocyclooctene lignans are called ‘Schisandra chinensis lignans’, even in the professional scientific literature [69].

*Schisandra Chinensis* (Turcz.) Baill. is the most famous plant in *Schisandraceae* family, and the fruits (called *Fructus Schizandrae*) were widely used as a traditional Chinese medicine for treating hepatitis, myocardial disorders, and hyperlipidemia and neurodegenerative diseases in the countries of East Asia and others [70,71,72]. In this plant, nine major bioactive lignans were identified as dibenzocyclooctenes, they are schisandrol A, schisandrol B, angeloylgomisin H, gomisin G, schisantherin A, schisanhenol, schisandrin A, schisandrin B, and schisandrin C [73]. In terms of antiviral activities, we have found that schisandrin A inhibits DENV replication via upregulating the antiviral interferon responses through the STAT signaling pathway [74]. Schisandrin A and schisandrin B exhibited antiviral activity against HIV [75], and schizandrin C was shown to be the most active compound in protection against liver injury in mice. A derivative of schizandrin C, Bicyclol, has been approved as a hepatoprotectant by the Chinese Food and Drug Administration (CFDA) for the treatment of liver injury in 2004 [76].

**Figure 9:**
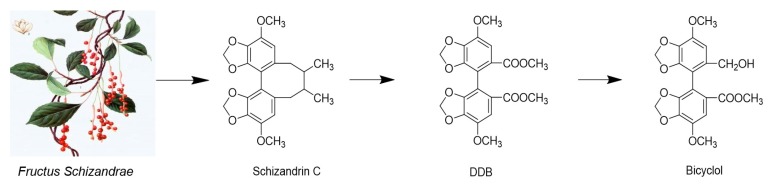
Bicyclol as an anti-HBV drug [76]. Schizandrin C was isolated from *F. Schizandrae* and verified as the most active compound in protection against liver injury in mice. DDB (Dimethyl dicarboxylate biphenyl) as an analog of schizandrin C has been widely used for the improvement of the abnormal liver function of CHB hepatitis in China. Bicyclol as a novel substitute for DDB was found to be more effective in protection against liver injury and was also showed to inhibit hepatitis virus replication in vitro and in vivo.

Bicyclol(4,4′-dimethoxy-5,6,5′,6′-bis(methylenedioxy)-2-hydroxymethyl-2′-methoxycarbonyl biphenyl) is an analog of the active component schizandrin C from *Fructus Schiznadrae* [77], as illustrated in Figure 9. Bicyclol was shown to have activities in vitro and in vivo. Clinical data showed that it could inhibit virus replication in patients that were infected with HBV, and the difference of the response to bicyclol therapy between HBV genotypes B and C was not statistically significant [78]. Other results showed that bicyclol significantly inhibited HCV replication in vitro and in hepatitis C patients [79]. Mechanistic studies suggest that anti-hepatitis activity of bicyclol is through the modulation of cytotoxic T lymphocytes [76], and by up-regulating the host restrictive factor (GLTP) for HCV replication and causing the spontaneous restriction of HCV replication [79]. Bicyclol is now used to treat the patients with chronic hepatitis B in China [80].

Rubrifloralignan A was isolated from another species of *Schisandraceae* family—*Schisandra rubriflora*. It can not only inhibits the formation of syncytium induced by HIV-1IIIB and cell death induced by HIV-1, but it also inhibits the replication of HIV. Mechanistically, rubrifloralignan A was shown to inhibit the early stage in HIV-1 replication [81]. The derivative, (+/−)-Gomisin M1, exhibited the most potent anti-HIV activity, with EC_50_ and SI values of <0.65 μM and >68, respectively [82]. Halogenated gomisin J derivatives were shown to be a nonnucleoside inhibitor of HIV type 1 reverse transcriptase [83].

### 2.2. Neolignans

**Figure 10:**
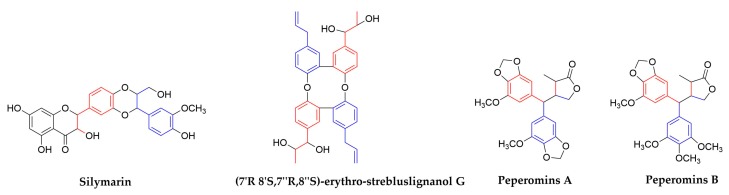
Structures of some neolignan compounds.

Neolignans are a class of lignans that do not contain the β-β′ (also referred to as an 8-8′) phenyl-propane linkage that are characteristic of classical lignans. They can be further grouped into different subtypes based on the nature and position of the linkage between the phenylpropane units. In contrast to classical lignans, there are only a few reports on the antiviral activities of neolignans. Figure 10 shows the structures of some neolignan compounds.

1,4-Benzodioxane lignans. This subtype of neolignans has received significant attention through the years due to their good biological activities. One representative is Silymarin flavonolignans, which were isolated from the seeds of *Silybum marianum* [84] and they are the most commonly consumed herbal products among the HCV-infected patients in western countries [85]. Besides, they were showed to possess antioxidative, anti-inflammatory, and hepatoprotective activities [86]. Recent studies have also documented the antiviral activities of silymarin and its derivatives against HCV and other viruses [87]. Its derivative intravenous silibinin, which was named Legalon^®^ SIL, and has been shown to block HCV production and increase anti-inflammatory and anti-proliferative gene expressions without affecting serum albumin levels in the clinical phase [88]. In addition, it was showed that silymarin inhibited the replication of IAV [89]. MOA showed that silymarin inhibited the late mRNA synthesis during IAV replication. It was also reported that silymarin inhibited other viruses, such as DENV, Chikungunya virus, Mayaro virus, HIV, and HBV [86].

(*7′R*,*8′S*,*7″R*,*8″S*)-erythro-strebluslignanol G, a neolignan and also a dimer of strebluslignanols, was isolated from the root of *Streblus asper*. It exhibits significant anti-HBV activities in the secretion of HBsAg and HBeAg, with IC_50_ values of 3.67 and 14.67 µM, respectively [90].

Secolignans or Cleavage lignans. These neolignans are presumed to be obtained by the pyrolysis, oxidation, and cyclization of arylnaphthalenes [91]. Most of the reported compounds were isolated from the plants of genus *Peperomia* (Piperaceae) [92], *Urtica* (Urticaceae) [93], and *Selaginella* (Selaginellaceae) [94]. They exhibited anti-tumor [95], anti-inflammatory [96], anti-HIV, insect antifeedant [97], and other pharmacological activities. Two compounds, Peperomins A and B, which were isolated from *Peperomia pellucida* (Piperaceae) [98], showed moderate inhibitory effects on HIV-1 IIIB growth in C8166 cells, with EC_50_ values of around 5 µM. However, it appears that the observed bioactivity was due to cytotoxicity [99].

## 3. Prospects of Lignans and Their Derivatives in Antiviral Development

Lignans are traditionally defined as a class of secondary metabolites that are derived from the oxidative dimerization of two or more phenylpropanoid units. They boast a vast structural diversity, despite their common biosynthetic origins. It is also well-established that this class of compounds exhibit a range of potent biological activities. Owing to these factors, lignans have proven to be a challenging and desirable synthetic target that have instigated the development of some different synthetic methods, advancing our collective knowledge towards the synthesis of complex and unique structures.

Virus-related diseases are becoming a more challenging public health concern with increased global travel and emergence of viral resistance to the clinical antiviral drugs. There is an urgent need to develop novel antiviral drugs targeting different viral and host proteins. Lignans, as discussed in this review, have large structural diversity and pharmacological activities, including antivirals. Two types of antiviral lignans—podophyllotoxin and bicyclol, which show high potency in the treatment of venereal warts and chronic hepatitis B, respectively—serve as good examples of developing lignans for antivirals. However, we believe that the potential of lignans in antivirals needs further exploration in the research and development. As noted above, although many of the classical lignans have been showed to display wide-range antiviral activities, little is known regarding the neolignans, which have more varied structures than classical lignans, with regards to their antiviral activities. These neolignans should be carefully evaluated to assess their activities against different viruses, and it is highly likely that many new antiviral activities will be discovered. Furthermore, action of mechanism studies should be investigated for facilitating the development of lead lignans in antiviral drug discovery.

## Figures and Tables

**Table 1:** The antiviral activities of lignans and their derivatives from plants.

Subclass	Cpd	From Plants	Organs	Virus(es)	IC_50_ (μM)	CC_50_ (μM)	Status	MOA/Targets	Refs
Dibenzylbutanes	Niranthin	*Phyllanthus niruri* L. (Euphorbiaceae)	Whole plants	HBV	15.6~25.1	369.9 In HepG 2.2.15	In Vitro In Vivo	inhibits DHBV DNA replication and HBV antigen expression.	[12,16]
	NDGA	*Larrea tridentate* (Zygophyllaceae)	Leaves (resin)	DENV	No data	No data	In Vitro	targets genome replication and viral assembly	[22,23,24,25]
				HCV	30	70 in Huh7		NDGA-mediated alterations of host lipid metabolism, LD morphology, and VLDL transport affect HCV proliferation	
				WNV/ZIKV	7.9/9.1	162.1 in Vero		WNV: disturb the lipid metabolism probably by interfering with the sterol regulatory element binding proteins (SREBP) pathway	
				IAV	In Vivo			suppresses replication of IAV and induction of cytokines, trypsin, and MMP-9, with improved animal survival	
	TMP	*Larrea tridentata* (Zygophyllaceae)	Leaves (resin)	WNV/ZIKV	9.3/5.7	1071.0 in Vero	In Vitro	impaires viral replication	[24,26,27,28,29,30]
				poxvirus	No data	No data	In Vitro	prevents the efficient spread of virus particles from cell to cell	
				HSV	43.5	160 in Vero	In Vitro	TMP inhibits both these viruses replication by blocking the binding of the host cell transcription factor, Sp1, to viral promoters.	
				HIV	25	No data	In Vitro		
				HPV	In Clinical			selectively interferes with HPV viral genes E6/E7 with Sp1dependent promoters, and induces apoptosis by inactivation of the CDC2/cyclin B complex (maturation promoting factor) and production and phosphorylation of survivin	
	Secoisolariciresinol dimethyleTher acetate	*Justicia procumbens* (Acanthaceae)	Air-dried aerial parts	HIV-1	5.27	11.6	In Vitro	waiting for the deeper research	[31]
Dibenzyltyrolactones	ATG	*Arctium lappa* L. (Compositae)	Whole plants	IAV	No data	No data	In Vitro In Vivo	induce the production of interferon	[32,33,34,35,36]
				HIV-1	No data	No data	In Vitro	inhibit the expression of protein P17 and P24 of the HIV-1 virus	
	Yatein	*Chamaecyparis obtuse* (Cupressaceae)	Dried leaves	HSV-1	30.6 ± 5.5	>100	In Vitro	inhibiting HSV-1 alpha gene expression, including expression of the ICP0 and ICP4 genes, and by arresting HSV-1 DNA synthesis and structural protein expression in HeLa cells	[37,38]
	Hinokinin	*Chamecyparis obtusa* (Cupressaceae)	Woods	HBV	No data	No data	In Vitro	waiting for the deeper research	[12,45,46,47]
				HIV	<28	527 in H9			
				SARS-CoV	>10	>750 in Vero			
				HCMV	No data	115 in A549			
Arylnaphthalenes	Diphyllin	genus *Haplophyllum* (Rutaceae)	Epigeal part	ZIKV	0.06	3.48 in MDCK	In Vitro	vacuolar ATPase (V-ATPase) inhibitors	[48,49,50,51,52]
				IAV	0.1–0.6 in different strains	24.1 in A549		inhibit endosomal acidification, thus interfering with downstream virus replication	
	DGP	*Justicia gendarussa* (Acanthaceae)	Stems and leaves	ZIKV	0.01–0.07	15–32	In Vitro In Vivo	prevented the acidification of endosomal/lysosomal compartments in target cells, thus inhibiting ZIKV fusion with cellular membranes and infection.	[51,53,54,55]
				HIV-1	15–21 nM	No data	In Vitro	HIV-1 reverse transcription	
Aryltetralins		*Dysosmae Verspiellis & Podophyllum peltatum* (Berberidaceae)	Roots and stems	Papilloma virus	Launched in China			waiting for the deeper research	[3,11,56,57,58]
Substituted tetrahydrofurans	lariciresinol-4-*O*-β-d-glucopyranoside	*Isatis indigotica* Fort (Cruciferae)	Roots	IAV	50 μg/mL	>200 μg/mL	In Vitro	pharmacological actions on the immune system, signal transduction, cell cycle, and metabolism	[62,63]
	(*7′R*, *8S*)-9′-lariciresinol-(α-methyl)-butanoate			HIV-1	0.66 mM	0.67mM in C8166	In Vitro	No report	[64]
	Isatindolignanoside A			CVB3	25.9	>100	In Vitro	waiting for the deeper research	[65]
	Clemastanin B			IAV	0.087–0.72 mg/mL	6.2–7.5 mg/mL	In Vitro	targets viral endocytosis, uncoating or RNP export from the nucleus	[66]
2,6-diarylfurofurans	Phillygenin	*Fructus Forsythiae* (Oleaceae)	Fruits	IAV	In Vivo			reduce inflammation caused by IAV.	[57,58]
	Sesamin	*Sesamum indicum* (Pedaliaceae)	Seeds	inflammatory cytokines induced by H1N1	No data	No data	In Vitro	anti-inflammatory cytokines in human PBMCs	[67]
Dibenzocyclooctene	Bicyclol	Analogue of schizandrin C from *Fructus Schiznadrae*		HBV	Launched in China			inhibit virus replication in patients infected with HBV	[76,77,78,79,80]
				HCV	30	No data	In vitro/Vivo/Clinical	modulation of cytotoxic T lymphocytes up-regulating the host restrictive factor (GLTP) for HCV replication, and causing spontaneous restriction of HCV replication	
	Rubrifloralignan A	*Schisandra rubriflora* (Schisandraceae)	Fruits	HIV-1	40.46	123.35	In Vitro	inhibit the early stage of HIV-1 replication	[81,82,83]
1,4-Benzodioxane lignans	Silymarin	*Silybum marianum* (Compositae)	Seeds	HCV	In Clinical			blocked HCV production, increased anti-inflammatory, anti-proliferative gene expressions without affecting serum albumin levels	[84,85,86,87,88,89]
				IAV	No data	No data	In Vitro	inhibition of late viral RNA synthesis	
Dimer of strebluslignanols	(*7′R*,*8′S*,*7″R*,*8″S*)-erythro-strebluslignanolG	*Streblus asper* (Moraceae)	Roots	HBV	3.67/HBsAg 14.67/HBeAg	No data	In Vitro	inhibit the secretion of HBsAg and HBeAg	[90]
Secolignans	Peperomins A&B	*Peperomia pellucida* (Piperaceae)	Whole plants	HIV-1 IIIB	5	No data	In Vitro	related to the cytotoxicity expressed as CC_50_ of compounds	[98,99]

IC_50_, inhibitory concentration of compound that produces 50% inhibition of virus-induced cytopathic effects; CC_50_, concentration that reduces the growth of target cells by 50%.

## References

[1] Teponno R.B., Kusari S., Spiteller M. (2016). Recent advances in research on lignans and neolignans. Nat. Prod. Rep..

[2] Ayres D.C., Loike J.D. (1991). Lignans. Chemical, Biological and Clinical Properties. Lignans Chem. Biol. Clin. Prop..

[3] Kaplan I.W. (1942). Condylomata acuminate. New Orleans Med. Surg. J..

[4] Wu X.Q., Li W., Chen J.X., Zhai J.W., Xu H.Y., Ni L., Wu S.S. (2019). Chemical Constituents and Biological Activity Profiles on Pleione (Orchidaceae). Molecules.

[5] Lu H.L., Liu G.T. (1992). Antioxidant activity of dibenzocyclooctene lignans isolated from *Schisandraceae*. Planta Med..

[6] Pan J.Y., Chen S.L., Yang M.H., Wu J., Sinkkonen J., Zou K. (2009). An update on lignans: Natural products and synthesis. Nat. Prod. Rep..

[7] Capilla A.S., Sánchez I., Caignard D.H., Renard P., Pujol M.D. (2001). Antitumor agents. Synthesis and biological evaluation of new compounds related to podophyllotoxin, containing the 2,3-dihydro-1,4-benzodioxin system. Eur. J. Med. Chem..

[8] Kawazoe K., Yutani A., Tamemoto K., Yuasa S., Shibata H., Higuti T., Takaishi Y. (2001). Phenylnaphthalene Compounds from the Subterranean Part of Vitex rotundifolia and Their Antibacterial Activity Against Methicillin-Resistant Staphylococcus aureus. J. Nat. Prod..

[9] Hirano T., Wakasugi A., Oohara M., Oka K., Sashida Y. (1991). Suppression of mitogen-induced proliferation of human peripheral blood lymphocytes by plant lignans. Planta Med..

[10] Iwasaki T., Kondo K., Kuroda T., Moritani Y., Yamagata S., Sugiura M., Kikkawa H., Kaminuma O., Ikezawa K. (1996). Novel selective PDE IV inhibitors as antiasthmatic agents. synthesis and biological activities of a series of 1-aryl-2,3-bis(hydroxymethyl)naphthalene lignans. J. Med. Chem..

[11] Charlton James L. (1998). Antiviral Activity of Lignans. J. Nat. Prod..

[12] Huang R.L., Huang Y.L., Ou J.C., Chen C.C., Hsu F.L., Chang C. (2003). Screening of 25 compounds isolated from *Phyllanthus* species for anti-human hepatitis B virus in vitro. Phytother Res..

[13] Pilkington L.I. (2018). Lignans: A Chemometric Analysis. Molecules.

[14] Kirkman L.M., Lampe J.W., Campbell D.R., Martini M.C., Slavin J.L. (1995). Urinary Lignan and isoflavonoid excretion in men and women consuming vegetable and soy diets. Nutr. Cancer.

[15] Jeffries D.E., Lindsley C.W. (2019). Asymmetric Synthesis of Natural and Unnatural Dibenzylbutane Lignans from a Common Intermediate. J. Org. Chem..

[16] Liu S., Wei W., Shi K., Cao X., Zhou M., Liu Z. (2014). In Vitro and in vivo anti-hepatitis B virus activities of the lignan niranthin isolated from *Phyllanthus niruri* L.. J. Ethnopharmacol..

[17] Hernandez D.J., Anderica R.A.C., Pedraza C.J. (2014). Paradoxical cellular effects and biological role of the multifaceted compound nordihydroguaiaretic acid. Arch. Pharm..

[18] Zúñiga-Toalá A., Zatarain-Barrón Z.L., Hernández-Pando R., Negrette-Guzmán M., Huerta-Yepez S., Torres I., Pinzón E., Tapia E., Pedraza-Chaverri J. (2013). Nordihydroguaiaretic acid induces Nrf2 nuclear translocation in vivo and attenuates renal damage and apoptosis in the ischemia and reperfusion model. Phytomedicine.

[19] Tong W., Ding X., Adrian T. (2002). The mechanisms of lipoxygenase inhibitor-induced apoptosis in human breast cancer cells. Biochem. Biophys. Res. Commun..

[20] Manzanero S., Santro T., Arumugam T.V. (2013). Neuronal oxidative stress in acute ischemic stroke: Sources and contribution to cell injury. Neurochem. Int..

[21] Floriano-Sanchez E., Villanueva C., Medina-Campos O.N., Rocha D., Sánchez-González D.J., Cárdenas-Rodríguez N., Pedraza-Chaverrí J. (2006). Nordihydroguaiaretic acid is a potent in vitro scavenger of peroxynitrite, singlet oxygen, hydroxyl radical, superoxide anion and hypochlorous acid and prevents in vivo ozone-induced tyrosine nitration in lungs. Free Radic Res..

[22] Soto A.R., Bautista C.P., Syed G.H., Siddiqui A., Del Angel R.M. (2014). Nordihydroguaiaretic acid (NDGA) inhibits replication and viral morphogenesis of dengue virus. Antivir. Res..

[23] Syed G.H., Siddiqui A. (2011). Effects of hypolipidemic agent nordihydroguaiaretic acid on lipid droplets and hepatitis C virus. Hepatology.

[24] Merino-Ramos T., de Oya N.J., Saiz J.-C., Martín-Acebes M.A. (2017). Antiviral Activity of Nordihydroguaiaretic Acid and Its Derivative Tetra-*O*-Methyl Nordihydroguaiaretic Acid against West Nile Virus and Zika Virus. Antimicrob. Agents Chemother..

[25] Wang S., Le T.Q., Kurihara N., Chida J., Cisse Y., Yano M., Kido H. (2010). Influenza virus-cytokine-protease cycle in the pathogenesis of vascular hyperpermeability in severe influenza. J. Infect. Dis..

[26] Oyegunwa A.O., Sikes M.L., Wilson J.R., Scholle F., Laster S.M. (2010). Tetra-*O*-methyl nordihydroguaiaretic acid (Terameprocol) inhibits the NF-kappaB-dependent transcription of TNF-alpha and MCP-1/CCL2 genes by preventing RelA from binding its cognate sites on DNA. J. Inflamm..

[27] Pollara J.J., Laster S.M., Petty I.T. (2010). Inhibition of poxvirus growth by Terameprocol, a methylated derivative of nordihydroguaiaretic acid. Antivir. Res..

[28] Chen H., Teng L., Li J.N., Park R., Mold D.E., Gnabre J., Hwu J.R., Tseng W.N., Huang R.C.C. (1998). Antiviral Activities of Methylated Nordihydroguaiaretic Acids. 2. Targeting Herpes Simplex Virus Replication by the Mutation Insensitive Transcription Inhibitor Tetra-*O*-methyl-NDGA. J. Med. Chem..

[29] Gnabre J.N., Brady J.N., Clanton D.J., Ito Y., Dittmer J., Bates R.B., Huang R.C. (1995). Inhibition of human immunodeficiency virus type 1 transcription and replication by DNA sequence-selective plant lignans. Proc. Natl. Acad. Sci. USA.

[30] Khanna N., Dalby R., Tan M., Arnold S., Stern J., Frazer N. (2007). Phase I/II clinical safety studies of terameprocol vaginal ointment. Gynecol. Oncol..

[31] Xu X., Wang D., Ku C., Zhao Y., Cheng H., Liu K.-L., Rong L.-J., Zhang H.-J. (2019). Anti-HIV lignans from *Justicia procumbens*. Chin. J. Nat. Med..

[32] Gao Y., Dong X., Kang T.G. (2002). Activity of in vitro anti-influenza virus of arctigenin. Chin. Herb. Med..

[33] Fu L., Xu P., Liu N., Yang Z., Zhang F., Hu Y. (2008). Antiviral effect of Arctigenin Compound on Influenza Virus. Tradit. Chin. Drug Res. Clin. Pharmacol..

[34] Hayashi K., Narutaki K., Nagaoka Y., Hayashi T., Uesato S. (2010). Therapeutic Effect of Arctiin and Arctigenin in Immunocompetent and Immunocompromised Mice Infected with Influenza A Virus. Biol. Pharm. Bull..

[35] Schröder H.C., Merz H., Steffen R., Müller W.E., Sarin P.S., Trumm S., Schulz J., Eich E. (1990). Differential in vitro anti-HIV activity of natural lignans. Zeitschrift für Naturforschung C.

[36] Eich E., Pertz H., Kaloga M., Schulz J., Pertz H., Eich E., Pommier Y. (1996). (−)-Arctigenin as a Lead Structure for Inhibitors of Human Immunodeficiency Virus Type-1 Integrase. J. Med. Chem..

[37] Kuo Y.C., Kuo Y.H., Lin Y.L., Tsai W.J. (2006). Yatein from Chamaecyparis obtusa suppresses herpes simplex virus type 1 replication in HeLa cells by interruption the immediate-early gene expression. Antivir. Res..

[38] Wang Y., Wang X., Xiong Y., Kaushik A.C., Muhammad J., Khan A., Dai H., Wei D.-Q. (2019). New strategy for identifying potential natural HIV-1 non-nucleoside reverse transcriptase inhibitors against drug-resistance: An in silico study. J. Biomol. Struct. Dyn..

[39] Chang C., Lien Y., Liu K.C.S.C., Li S.-S. (2003). Lignans from *Phyllanthus urinaria*. Phytochemistry.

[40] Kuo P., Li Y., Wu T. (2012). Chemical Constituents and Pharmacology of the Aristolochia species. J. Tradit. Complement. Med..

[41] Gangan V., Hussain S.S. (2011). Alkaloids from Piper hookeri: Revision of NMR assignments by the application of 2D NMR spectroscopy. J. Pharm. Res..

[42] Nunomura S., Yoshida M. (2002). Lignans and benzoic acid derivatives from pericarps of Virola multinervia (Myristicaceae). Biochem. Syst. Ecol..

[43] Schmidt T.J., Hemmati S., Klaes M., Konuklugil B., Mohagheghzadeh A., Ionkova I., Fuss E., Alfermann A.W. (2010). Lignans in flowering aerial parts of Linum species—chemodiversity in the light of systematics and phylogeny. Phytochemistry.

[44] Marcotullio M.C., Pelosi A., Curini M. (2014). Hinokinin, an emerging bioactive lignan. Molecules.

[45] Cheng M.-J., Lee K.-H., Tsai I.-L., Chen I.-S. (2005). Two new sesquiterpenoids and anti-HIV principles from the root bark of Zanthoxylum ailanthoides. Bioorg. Med. Chem..

[46] Wen C.-C., Kuo Y.-H., Jan J.-T., Liang P.-H., Wang S.-Y., Liu H.-G., Li C.-K., Chang S.-T., Kuo C.-J., Lee S.-S. (2007). Specific Plant Terpenoids and Lignoids Possess Potent Antiviral Activities against Severe Acute Respiratory Syndrome Coronavirus. J. Med. Chem..

[47] Rozália P., Abrantes M., Serly J., Duarte N., Molnar J., Ferreira M.-J.U. (2010). Antitumor-promoting Activity of Lignans: Inhibition of Human Cytomegalovirus IE Gene Expression. Anticancer Res..

[48] Chen H., Liu P., Zhang T., Gao Y., Zhang Y., Shen X., Li X., Shen W. (2018). Effects of diphyllin as a novel V-ATPase inhibitor on TE-1 and ECA-109 cells. Oncol. Rep..

[49] Nesmelova E.F., Razakova D.M., Akhmedzhanova V.I., Bessonova I.A. (1983). Diphyllin from *Haplophyllum alberti-regelii*, *H. bucharicum*, and *H. perforatum*. Chem. Nat. Compd..

[50] Sørensen M.G., Henriksen K., Neutzsky-Wulff A.V., Dziegiel M.H., Karsdal M.A. (2007). Diphyllin, a Novel and Naturally Potent V-ATPase Inhibitor, Abrogates Acidification of the Osteoclastic Resorption Lacunae and Bone Resorption. J. Bone Miner. Res. Off. J. Am. Soc. Bone Miner. Res..

[51] Martinez-Lopez A., Persaud M., Chavez M.P., Zhang H., Rong L., Liu S., Wang T.T., Sarafianos S.G., Diaz-Griffero F. (2019). Glycosylated diphyllin as a broad-spectrum antiviral agent against Zika virus. EBioMedicine.

[52] Chen H.-W., Cheng J.X., Liu M.-T., King K., Peng J.Y., Zhang X.-Q., Wang C.-H., Shresta S., Schooley R.T., Liu Y.-T. (2013). Inhibitory and combinatorial effect of diphyllin, a v-ATPase blocker, on influenza viruses. Antivir. Res..

[53] Susplugas S., Hung N., Bignon J., Thoison O., Kruczynski A., Sévenet T., Guéritte F. (2005). Cytotoxic Arylnaphthalene Lignans from a Vietnamese Acanthaceae. *Justicia patentiflora*. J. Nat. Prod..

[54] Zhang H.-J., Rumschlag-Booms E., Guan Y.-F., Liu K.-L., Wang D.-Y., Li W.-F., Nguyen V.H., Cuong N.M., Soejarto D.D., Fong H.H.S. (2017). Anti-HIV diphyllin glycosides from Justicia gendarussa. Phytochemistry.

[55] Zhang H.-J., Rumschlag-Booms E., Guan Y.-F., Wang D.-Y., Liu K.-L., Li W.-F., Nguyen V.H., Cuong N.M., Soejarto D.D., Fong H.H.S. (2017). Potent Inhibitor of Drug-Resistant HIV-1 Strains Identified from the Medicinal Plant Justicia gendarussa. J. Nat. Prod..

[56] Zalesak F., Bon D.J.D., Pospisil J. (2019). Lignans and Neolignans: Plant secondary metabolites as a reservoir of biologically active substances. Pharm. Res..

[57] Alsdorf W., Seidel C., Bokemeyer C., Oing C. (2019). Current pharmacotherapy for testicular germ cell cancer. Expert Opin. Pharm..

[58] Komericki P., Akkilic-Materna M., Strimitzer T., Aberer W. (2011). Efficacy and Safety of Imiquimod Versus Podophyllotoxin in the Treatment of Anogenital Warts. Sex. Transm. Dis..

[59] Ma Z.-J., Lu L., Yang J.-J., Wang X.-X., Su G., Wang Z., Chen G., Sun H., Wang M., Yang Y. (2018). Lariciresinol induces apoptosis in HepG2 cells via mitochondrial-mediated apoptosis pathway. Eur. J. Pharm..

[60] Zhao D., Wu T.Y., Guan Y.Q., Ma G.X., Zhang J., Shi L.L. (2017). Chemical constituents from roots of *Stelleropsis tianschanica*. China J. Chin. Mater. Med..

[61] Bajpai V.K., Shukla S., Paek W.K., Lim J., Kumar P., Kumar P., Na M.K. (2017). Efficacy of (+)-Lariciresinol to Control Bacterial Growth of Staphylococcus aureus and Escherichia coli O157:H7. Front. Microbiol..

[62] Li J., Zhou B., Li C., Chen Q.Y., Wang Y., Li Z., Chen T., Yang C., Jiang B., Zhong Z. (2015). Lariciresinol-4-*O*-beta-d-glucopyranoside from the root of *Isatis indigotica* inhibits influenza A virus-induced pro-inflammatory response. J. Ethnopharmacol..

[63] Zhou B., Li J., Liang X., Yang Z., Jiang Z. (2017). Transcriptome profiling of influenza A virus-infected lung epithelial (A549) cells with lariciresinol-4-beta-D-glucopyranoside treatment. PLoS ONE.

[64] Liu Z.L., Liu Y.Q., Zhao L., Xu J., Tian X. (2010). The phenylpropanoids of Aster flaccidus. Fitoterapia.

[65] Meng L., Guo Q., Chen M., Jiang J., Li Y., Shi J. (2018). Isatindolignanoside A, a glucosidic indole-lignan conjugate from an aqueous extract of the *Isatis indigotica* roots. Chin. Chem. Lett..

[66] Yang Z., Wang Y., Zheng Z., Zhao S., Zhao J., Lin Q., Li C., Zhu Q., Zhong N. (2013). Antiviral activity of *Isatis indigotica* root-derived clemastanin B against human and avian influenza A and B viruses in vitro. Int. J. Mol. Med..

[67] Fanhchaksai K., Kodchakorn K., Pothacharoen P., Kongtawelert P. (2016). Effect of sesamin against cytokine production from influenza type A H1N1-induced peripheral blood mononuclear cells: Computational and experimental studies. Vitr. Cell Dev. Biol. Anim..

[68] Ren R., Ci X.-X., Li H.-Z., Luo G.-J., Li R.-T., Deng X.-M. (2014). New Dibenzocyclooctadiene Lignans from Schisandra sphenanthera and Their Proinflammatory Cytokine Inhibitory Activities. Z. Für Nat. B.

[69] Szopa A., Barnaś M., Ekiert H. (2018). Phytochemical studies and biological activity of three Chinese Schisandra species (Schisandra sphenanthera, Schisandra henryi and Schisandra rubriflora): Current findings and future applications. Phytochem. Rev..

[70] Checker R., Patwardhan R., Sharma D., Menon J., Thoh M., Bhilwade H.N., Konishi T., Sandur S.K. (2012). Schisandrin B exhibits anti-inflammatory activity through modulation of the redox-sensitive transcription factors Nrf2 and NF-kappaB. Free Radic. Biol. Med..

[71] Park S.Y., Park S.J., Park T.G., Rajasekar S., Lee S.-J., Choi Y.W. (2013). Schizandrin C exerts anti-neuroinflammatory effects by upregulating phase II detoxifying/antioxidant enzymes in microglia. Int. Immunopharmacol..

[72] Szopa A., Ekiert R., Ekiert H. (2017). Current knowledge of Schisandra chinensis (Turcz.) Baill. (Chinese magnolia vine) as a medicinal plant species: A review on the bioactive components, pharmacological properties, analytical and biotechnological studies. Phytochem. Rev..

[73] Liu H., Lai H., Jia X., Liu J., Zhang Z., Qi Y., Zhang J., Song J., Wu C., Zhang B. (2013). Comprehensive chemical analysis of Schisandra chinensis by HPLC-DAD-MS combined with chemometrics. Phytomed. Int. J. Phytother. Phytopharm..

[74] Yu J.-S., Wu Y.-H., Tseng C.-K., Lin C.-K., Hsu Y.-C., Chen Y.-H., Lee J.-C. (2017). Schisandrin A inhibits dengue viral replication via upregulating antiviral interferon responses through STAT signaling pathway. Sci. Rep..

[75] Xu L., Grandi N., Del Vecchio C., Mandas D., Corona A., Piano D., Esposito F., Parolin C., Tramontano E. (2015). From the traditional Chinese medicine plant Schisandra chinensis new scaffolds effective on HIV-1 reverse transcriptase resistant to non-nucleoside inhibitors. J. Microbiol..

[76] Liu G. (2009). Bicyclol: A Novel Drug for Treating Chronic Viral Hepatitis B and C. Med. Chem..

[77] Zhang T. (2016). New drugs derived from medicinal plants. Thérapie.

[78] Ruan B., Wang J., Bai X. (2007). Comparison of bicyclol therapy for patients with genotype B and C of hepatitis B virus. Chin. J. Exp. Clin. Virol..

[79] Huang M.-H., Li H., Xue R., Li J., Wang L., Cheng J., Wu Z., Li W., Chen J., Lv X. (2019). Up-regulation of glycolipid transfer protein by bicyclol causes spontaneous restriction of hepatitis C virus replication. Acta Pharm. Sin. B.

[80] Zhou Y., Chai X. (2019). Protective effect of bicyclol against pulmonary fibrosis via regulation of microRNA5 in rats. J. Cell. Biochem..

[81] Tian R.R., Xiao W.L., Yang L.M., Wang R.R., Sun H.D., Liu N.F., Zheng Y.T. (2006). The Isolation of Rubrifloralignan A and Its Anti-HIV-1 Activities. Chin. J. Nat. Med..

[82] Chen M., Kilgore N., Lee K.-H., Chen D.-F. (2006). Rubrisandrins A and B, Lignans and Related Anti-HIV Compounds from Schisandra rubriflora. J. Nat. Prod..

[83] Fujihashi T., Hara H., Sakata T., Mori K., Higuchi H., Tanaka A., Kaji H., Kaji A. (1995). Anti-human immunodeficiency virus (HIV) activities of halogenated gomisin J derivatives, new nonnucleoside inhibitors of HIV type 1 reverse transcriptase. Antimicrob. Agents Chemother..

[84] Federico A., Dallio M., Loguercio C. (2017). Silymarin/Silybin and Chronic Liver Disease: A Marriage of Many Years. Molecules.

[85] Strader D.B., Bacon B.R., Lindsay K.L., La Brecque D.R., Morgan T., Wright E.C., Seeffff L.B. (2002). Use of complementary and alternative medicine in patients with liver disease. Am. J. Gastroenterol..

[86] Liu C.-H., Jassey A., Hsu H.-Y., Lin L.-Z. (2019). Antiviral Activities of Silymarin and Derivatives. Molecules.

[87] Wagoner J., Negash A., Kane O.J., Martinez L.E., Nahmias Y., Bourne N., Owen D.M., Grove J., Brimacombe C., McKeating J.A. (2010). Multiple effects of silymarin on the hepatitis C virus lifecycle. Hepatology.

[88] DebRoy S., Hiraga N., Imamura M., Hayes C.N., Akamatsu S., Canini L., Perelson A.S., Pohl R.T., Persiani S., Uprichard S.L. (2016). Hepatitis C virus dynamics and cellular gene expression in uPA-SCID chimeric mice with humanized livers during intravenous silibinin monotherapy. J. Viral Hepat..

[89] Song J.H., Choi H.J. (2011). Silymarin efficacy against influenza a virus replication. Phytomedicine.

[90] Li J., Meng A.-P., Guan X.-L., Li J., Wu Q., Deng S.-P., Su X.-J., Yang R.-Y. (2013). Anti-hepatitis B virus lignans from the root of Streblus asper. Bioorg. Med. Chem. Lett..

[91] Lu X., Zhang W., Cheng X.-H., Wang H.-G., Yu D.-Y., Feng B.-M. (2014). Advances on the secolignans compounds in natural products. J. Shenyang Pharm. Univ..

[92] Su X., Na L., Ning M.-M., Zhou C.-H., Yang Q.-R., Wang M.W. (2006). Bioactive Compounds from Peperomia pellucida. J. Nat. Prod..

[93] Feng B.M., Qin H.H., Wang H.G., Shi L.Y., Yu D.Y., Ji B.Q., Zhao Q., Wang Y.Q. (2012). Three new secolignan glycosides from Urtica fissa E. Pritz. J. Nat. Med..

[94] Feng W.-S., Chen H., Zheng X.-K., Wang Y.-Z., Chen H., Li Z. (2009). Two new secolignans from Selaginella sinensis (Desv.) Spring. J. Asian Nat. Prod. Res..

[95] Cheng M.-J., Lee S.-J., Chang Y.-Y., Wu S.-H., Tsai I.-L., Jayaprakasam B., Chen I.-S. (2003). Chemical and cytotoxic constituents from Peperomia sui. Phytochemistry.

[96] Tsutsui C., Yamada Y., Ando M., Toyama D., Wu J.L., Wang L., Taketani S., Kataoka T. (2009). Peperomins as anti-inflammatory agents that inhibit the NF-kappaB signaling pathway. Bioorg. Med. Chem. Lett..

[97] Govindachari T.R., Kumari G.N.K., Partho P.D. (1998). Two secolignans from Peperomia dindigulensis. Phytochemistry.

[98] Lin M., Yu D., Wang Q. (2011). Secolignans with Antiangiogenic Activities from Peperomia dindygulensis. Chem. Biodivers..

[99] Zhang G.-L., Li N., Wang Y.-H., Zheng Y.-T., Zhang Z., Wang M.-W. (2007). Bioactive lignans from Peperomia heyneana. J. Nat. Prod..

